# The real-world safety of oseltamivir and baloxavir marboxil in children: a disproportionality analysis of the FDA adverse event reporting system

**DOI:** 10.3389/fphar.2024.1391003

**Published:** 2024-07-10

**Authors:** Wei Wei, Liang Huang, Yingtao Bai, En Chang, Jinfeng Liu

**Affiliations:** ^1^ Department of Pharmacy, People’s Hospital of Zhongjiang County, Deyang, China; ^2^ Department of Pharmacy and Evidence-Based Pharmacy Center, West China Second University Hospital, Chengdu, China; ^3^ Key Laboratory of Birth Defects and Related Diseases of Women and Children (Sichuan University), Ministry of Education, Chengdu, China

**Keywords:** oseltamivir, baloxavir marboxil, FAERS, pharmacovigilance, signal detection

## Abstract

**Background:**

Oseltamivir and baloxavir marboxil are the two primary oral drugs approved by the Food and Drug Administration (FDA) for treating influenza. Limited real-world evidence exists on their adverse events in children. The purpose of this study was to explore the adverse event (AE) profiles of oseltamivir and baloxavir marboxil in children based on the U.S. Food and Drug Administration Adverse Event Reporting System (FAERS) database.

**Methods:**

FAERS reports were collected and analyzed from the first quarter of 2019 to the third quarter of 2023. Disproportionality analyses, including the reporting odds ratio (ROR), the proportional reporting ratio (PRR), the Bayesian confidence propagation neural network (BCPNN), and the multi-item gamma Poisson shrinker (MGPS) algorithms, were employed in data mining to quantify the signals of oseltamivir and baloxavir marboxil-related AEs.

**Results:**

A total of 464 reports of AEs to oseltamivir as the “primary suspect (PS)” and 429 reports of AEs to baloxavir marboxil as the “PS” were retrieved in pediatric patients. A total of 100 oseltamivir-induced AE signals were detected in 17 system organ classes (SOCs), and 11 baloxavir marboxil-induced AE signals were detected in 6 SOCs after complying with the four algorithms simultaneously. Categorized and summarized by the number of reports of involvement in each SOC, the top 3 for oseltamivir were psychiatric disorders, gastrointestinal disorders, general disorders and site-of-administration conditions, respectively. The top 3 for baloxavir marboxil were injury, poisoning and surgical complications, general disorders and site of administration conditions, and psychiatric disorders, respectively.

**Conclusion:**

Our study identifies potential new AE signals for oseltamivir and provides a broader understanding of the safety of oseltamivir and baloxavir marboxil in children.

## 1 Introduction

Acute respiratory diseases are common in children, and one of the common pathogens of influenza virus (Heikkinen, 2006). Influenza virus infections can occur in children of all ages from birth to 18 years of age (Fowlkes et al., 2014; Caini et al., 2018). While this condition is typically self-limiting in healthy adults, it may lead to severe complications in children (Paules and Subbarao, 2017). The younger the child, the sicker the flu and the higher the rate of complications (Principi and Esposito, 2016; Uyeki, 2020). In addition, the high incidence of seasonal influenza in school-age children has led to a significant spread of influenza viruses (Rotrosen and Neuzil, 2017; Nayak, Hoy, and Gordon, 2021), and has imposed a socioeconomic burden on affected children and families (Principi et al., 2003; Ambrose and Antonova, 2014; McLean et al., 2017). And anti-influenza viral drugs have been shown to be effective in preventing and treating influenza by reducing the duration and severity of illness (Fiore et al., 2011).

Oseltamivir and baloxavir marboxil are the two most commonly used oral agents approved by the FDA for the treatment of influenza. Oseltamivir is a neuraminidase inhibitor (NAI) that prevents the influenza virus from replicating and reduces infectiousness. It effectively shortens the time to symptom relief (Jefferson et al., 2014), hospitalization rates, and the incidence of complications in pediatric influenza patients (Peters et al., 2008). Oseltamivir is a first-line drug for the prevention and treatment of influenza (Committee on Infectious Diseases, 2023). Baloxavir marboxil is the first FDA approval of the world’s first anti-influenza virus drug based on a completely new mechanism in nearly 20 years. It was approved for marketing in Japan and the United States in 2018. Baloxavir marboxil is a novel cap-dependent endonuclease inhibitor. Unlike neuraminidase inhibitors that block viral release from infected host cells, baloxavir marboxil blocks influenza virus proliferation by inhibiting viral messenger Ribonucleic Acid (mRNA) transcription (F.G. Hayden et al., 2018).

However, there needs to be more data evaluating the safety of oseltamivir and baloxavir marboxil in children, and findings on the safety of oseltamivir and baloxavir are inconsistent. A meta-analysis that included three randomized controlled trials (RCTs) noted that baloxavir marboxil appears to be a relatively safe anti-influenza drug compared to oseltamivir (Kuo et al., 2021). In contrast, a recent study, which included 200 patients aged 14–85 years with a diagnosis of influenza A, showed that the difference in the incidence of adverse events between the two groups of patients treated with baloxavir marboxil and oseltamivir was not statistically significant (Qiu et al., 2024). These discrepancies highlight the need for more comprehensive safety evaluations, particularly in pediatric populations. Clinical trial results may not always reflect real-world outcomes (Ma et al., 2021), and women are increasingly acknowledged as a risk factor for significant side effects with clinical relevance (Franconi and Campesi, 2014). Therefore, there is an urgent need for pharmacovigilance studies to examine the adverse reaction profiles of oseltamivir and baloxavir marboxil in children.

The FAERS database is a valuable resource for post-marketing surveillance and early detection of drug safety problems (Feng et al., 2022). Although it is impossible to explain the causal relationship between drugs and AEs, disproportionality analysis in the spontaneous reporting of adverse drug reactions database remains a validated quantitative methodology for pharmacovigilance signal detection (Cutroneo et al., 2024). In-depth analysis of the FAERS data in this study allowed for a comprehensive assessment of the safety of oseltamivir and baloxavir marboxil in a real-world pediatric population, thereby increasing clinical awareness, enhancing proactive surveillance, and promoting safer medication use.

## 2 Materials and methods

### 2.1 Data sources

The FAERS database is a free U.S. database where health professionals, consumers, manufacturers, and others may voluntarily submit AE reports to help the FDA monitor the safety of medicines and biologics after they have been marketed (Cirmi et al., 2020; Hu et al., 2020). FAERS has been noted for its capability to identify early safety concerns, especially for recently authorized medications and uncommon adverse reactions (Harpaz et al., 2013; Fukazawa et al., 2018). The FAERS database is updated every 3 months, and anybody may freely access and download the data from the FDA website. The FAERS database contains seven modules corresponding to seven aspects of the main content, including patient demographic and administrative information (DEMO), report source (RPSR), drug information (DRUG), adverse events (REAC), patient outcomes (OUTC), indications for drug administration (INDI), and therapy start states and end dates for the reported drugs (THER). In this study, data on baloxavir marboxil and oseltamivir were extracted from the FAERS database for the period January 2019 to September 2023 (the most recent data available in the FAERS database) and all data were imported into MySQL 8.0 for analysis.

**FIGURE 1:**
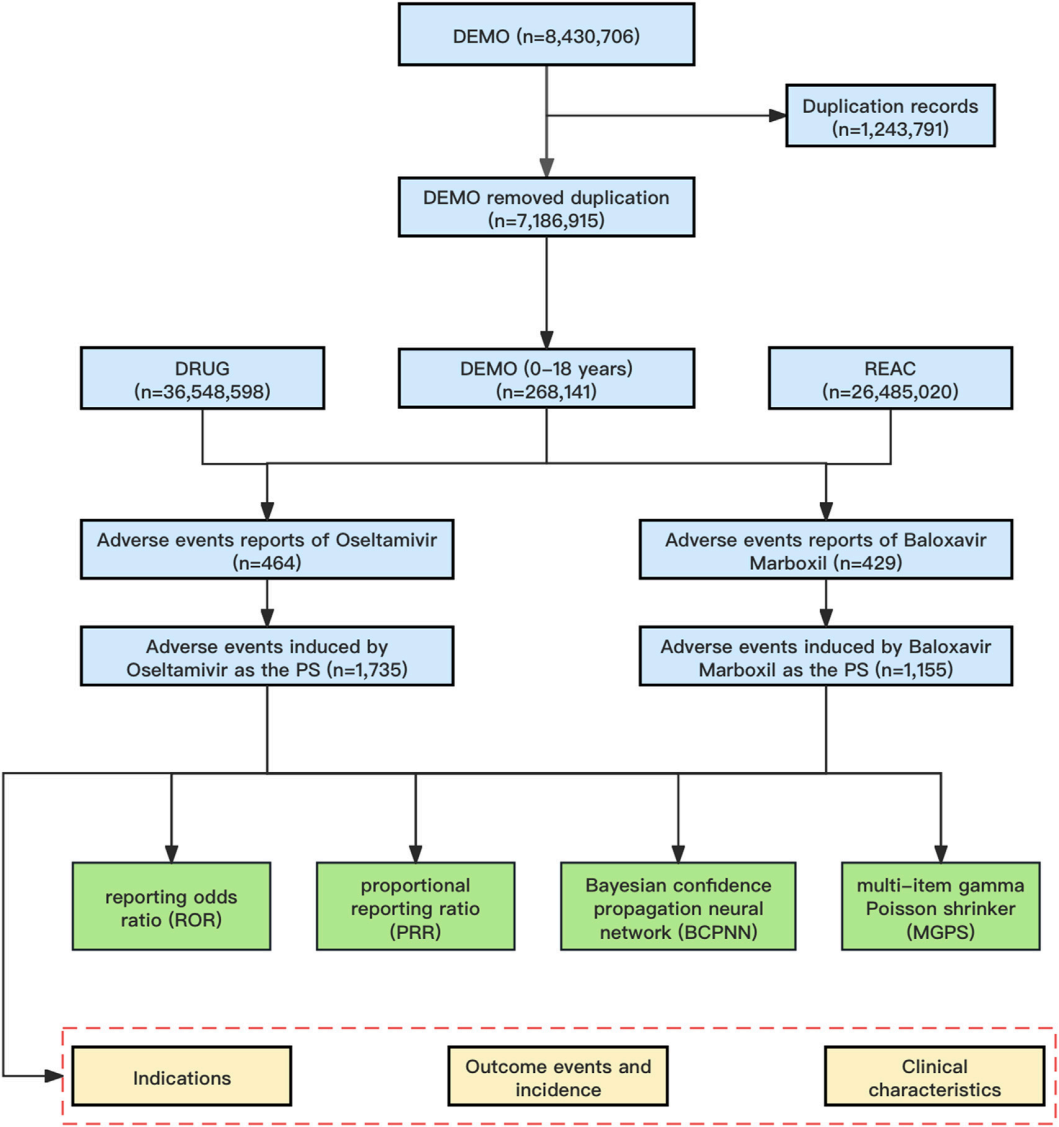
The flowchart of identifying oseltamivir and baloxavir marboxil AEs in FAERS database. Abbreviations: FAERS, United States Food and Drug Administration Adverse Event Reporting System; DEMO, demographic and administrative information file; DRUG, drug information file; REAC, adverse events file; PS, Primary Suspect.

A total of 8,430,706 AE reports were retrieved from the FAERS database. We used a two-step deduplication process to ensure the uniqueness of the reports. First, we downloaded the deleted data from the deleted files from the FAERS database, and then performed the deduplication process according to the FDA recommendations by selecting the higher primaryid when the caseid and FDA_DT are the same, and selecting the most recent FDA_DT when the caseid are the same (Sakaeda et al., 2013), which ultimately reduced the number of reports to 7,186,915 (Figure 1). We further filtered the dataset to include only those reports in which oseltamivir or baloxavir marboxil was the PS drug. This means that in all of our study’s AE reports, oseltamivir or baloxavir marboxil were the only drugs coded as “PS” in the role_cod field. This improves the reliability of the study results.

### 2.2 Procedures

We used FDA-approved generic and trade names to identify AEs in Drug files by fuzzy matching, including oseltamivir (TAMIFLU) AND baloxavir marboxil (XOFLUZA). To improve the accuracy of the analysis, we restricted the analysis to reports with a drug role_cod of “PS (primary suspect)” in the Drug file. AEs in FAERS are coded by the preferred term (PT) in the Standardized Medical Dictionary (MedDRA) terminology (Brown et al., 1999), which is structured at five levels: system organ class (SOC), high level group term (HLGT), high level term (HLT), PT, and lowest level term (LLT). In addition, different PTs can be combined to define specific clinical syndromes using an algorithm known as a standardized MedDRA query.

### 2.3 Data mining

Disproportionality analysis is a key tool in pharmacovigilance research used to detect medication-related AEs by comparing the occurrence of AEs between a certain medicine and all other drugs (Hu et al., 2020). The general principle is that a meaningful signal is considered to be generated when the incidence of a specific AE for a particular drug is significantly higher than the background frequency in the database and reaches a certain threshold or criterion. The study utilized four algorithms: proportional reporting ratio (PRR), reporting odds ratio (ROR), Bayesian confidence propagation neural network (BCPNN), and multi-item gamma Poisson shrinker (MGPS) (van Puijenbroek et al., 2002; Song et al., 2020). PRR and ROR are examples of frequencyist approaches, which do not use Bayesian principles. On the other hand, BCPNN and MGPS are Bayesian methods (Sakaeda et al., 2013). Information Components (IC) are utilized within the BCPNN tool to quantify disproportionality (Bate, 2007). The utilization of MGPS analysis is a well-acknowledged approach for mitigating the occurrence of inaccurate positive findings. It accomplishes this by employing a Bayesian shrinkage estimator to modify the observed/expected ratio. This adjustment results in smaller risk estimates with narrower confidence intervals, even when the event counts are low (Napoli et al., 2014). The two Bayesian approaches, BCPNN and MGPS, were deemed valuable because of their ability to identify distinct signals, even in cases where there were few reports of AEs for a specific medication (Nomura et al., 2015). In general, an increase in the value of the four parameters leads to a corresponding increase in the strength of the signal value. The precise formulas and criteria used by the four algorithms for detecting positive safety signals were deemed valuable. The presence of the four algorithms is demonstrated in Table 1. To ensure the findings’ dependability, we selected the AE signals that meet the thresholds of all four methods simultaneously for the investigation.

**TABLE 1:** Four major algorithms used for signal detection.

Algorithms	Equation	Criteria
ROR	ROR=ad/b/c	lower limit of 95% CI > 1, N ≥ 3
	$95\%\text{CI}=e^{\ln\left(\text{ROR}\right)\pm 1.96(1/a+1/b+1/c+1/d\hat{)}0.5}$	
PRR	$\text{PRR}=a\left(c+d\right)/c/\left(a+b\right)$	PRR≥2, χ^2^ ≥ 4, N ≥ 3
	$\chi^{2}=\left[\left(\text{ad}-\text{bc}\hat{)}2\right.\right]\left(a+b+c+d\right)/\left[\left(a+b\right)\left(c+d\right)\left(a+c\right)\left(b+d\right)\right]$	
BCPNN	$\text{IC}=\log_{2}a\left(a+b+c+d\right)/\left(a+c\right)/\left(a+b\right)$	IC025 > 0, N ≥ 3
	$95\%\text{CI}=E\left(\text{IC}\right)\;\pm \;2V(\text{IC}\hat{)}0.5$	
	$r=(a+b+c+d\hat{)}2/\left(a+b+1\right)/\left(a+c+1\right)$	
	$E\left(\text{IC}\right)=\log_{2}a(a+b+c+d\hat{)}2/\left(a+b+c+d+r\right)/\left(a+b\right)/\left(a+c\right)$	
	$V\left(\text{IC}\right)=1/\ln2\left\{\left(b+c+d+r-1\right)/\left(a+1\right)/\left(a+b+c+d+r+1\right)+\left(2+b+c+2d\right)/\left(a++b+1\right)/\left(a+b+c+d+r+3\right)\right\}$	
	$\text{IC}025=E\left(\text{IC}\right)-\;2V(\text{IC}\hat{)}0.5$	
MGPS	$\text{EBGM}=a\left(a+b+c+d\right)/\left(a+c\right)/\left(a+b\right)$	EBGM05 ≥ 2, N ≥ 3
	$95\%\text{CI}=e^{\ln\left(\text{EBGM}\right)\pm 1.96(1/a+1/b+1/c+1/d\hat{)}0.5}$	
	$\text{EBGM}05=e^{\ln\left(\text{EBGM}\right)-1.96(1/a+1/b+1/c+1/d\hat{)}0.5}$	

Equation: a, number of reports containing both the suspect drug and the suspect adverse drug reaction; b, number of reports containing the suspect adverse drug reaction with other medications (except the drug of interest); c, number of reports containing the suspect drug with other adverse drug reactions (except the event of interest); d, number of reports containing other medications and other adverse drug reactions. ROR, reporting odds ratio; CI, confidence interval; N, the number of co-occurrences; PRR, proportional reporting ratio; χ^2^, chi-squared; BCPNN, bayesian confidence propagation neural network; IC, information component; IC025, the lower limit of the 95% one-sided CI, of the IC; MGPS, multi-item gamma Poisson shrinker; EBGM, empirical Bayesian geometric mean; EBGM05, the lower 95% one-sided CI, of EBGM.

### 2.4 Subgroup analysis

We performed subgroup analyses according to the gender of the patients (female and male) to study the differences in AEs due to oseltamivir *versus* baloxavir marboxil across different genders.

## 3 Results

### 3.1 Descriptive analysis

From January 2019 through September 2023, a total of 8,430,706 AE reports were recorded in the FAERS database, which was reduced to 7,186,915 AE reports by removing duplicates, and then further screened to identify 268,141 AE reports aged 0–18 years. In the present study, 464 reports identified oseltamivir as the PS, corresponding to a total of 1,735 AEs attributed to oseltamivir. Similarly, for baloxavir marboxil, 429 reports identified it as the PS, with a total of 1,155 AEs linked to baloxavir marboxil as the suspected drug. The mean age of pediatric patients treated with oseltamivir was 8.28 years while the mean age of patients treated with baloxavir marboxil was 10.52 years. The highest number of AEs were reported in the age group of 6–12 years with 187 (40.30%) for oseltamivir and 322 (75.06%) for baloxavir marboxil. In addition, the highest number of reports from consumers were 267 cases (57.54%) for oseltamivir and 279 cases (65.03%) for baloxavir marboxil. As for the countries where the adverse events were reported, the countries with the highest number of reports were all from the United States, which were oseltamivir 295 cases (63.58%) and baloxavir marboxil 320 cases (74.59%), respectively. See Table 2 for details. 194 patients treated with oseltamivir reported 579 concomitant medications, while 69 patients treated with baloxavir marboxil reported 156 concomitant medications. Table 3 lists concomitant medications with oseltamivir and baloxavir marboxil (the top five). The most used concomitant drugs were all acetaminophen.

**TABLE 2:** Clinical characteristics of reports with oseltamivir and baloxavir marboxil from the FAERS database (January 2019 to September 2023).

Characteristics	Subgroups	Oseltamivir		Baloxavir	
		Case number, n	Case proportion, %	Case number, n	Case proportion, %
Number of events		464		429	
Gender	Female	197	42.46	169	39.39
	Male	249	53.66	182	42.42
	Unknown	18	3.88	78	18.18
Age	Mean ± SD	8.28 ± 5.07		10.52 ± 3.12	
	0–1 year	51	10.99	4	0.93
	2–5 years	113	24.35	25	5.83
	6–12 years	187	40.30	322	75.06
	13–18 years	113	24.35	79	18.41
Reporter	Physician	67	14.44	106	24.71
	Pharmacist	110	23.71	32	7.46
	Other health-professional	15	3.23	12	2.80
	Consumer	267	57.54	279	65.03
	Lawyer	2	0.43	0	0.00
	Unknown	3	0.65	0	0.00
Reported Countries	America	295	63.58	320	74.59
	China	28	6.03	2	0.47
	Japan	26	5.60	105	24.48
	Germany	15	3.23	0	0.00
	Others and country not specified	100	21.55	2	0.47
Year	2023q1-q3	24	5.17	11	2.56
	2022	38	8.19	25	5.83
	2021	35	7.54	4	0.93
	2020	156	33.62	256	59.67
	2019	212	45.69	134	31.24
Indications	Influenza	311	67.03	166	38.69
	Others and blank	153	32.97	263	61.31
Serious Outcome	Death	27	5.82	3	0.70
	Disability	9	1.94	1	0.23
	Hospitalization	72	15.52	48	11.19
	Life-Threatening	16	3.45	6	1.40
	Other Serious (Important Medical Event)	175	37.72	56	13.05
	Required Intervention to Prevent Permanent Impairment/Damage	3	0.65	0	0.00
	Unknown	162	34.91	315	73.43

Abbreviations: FAERS, united states food and drug administration adverse event reporting system; SD, standard deviation; q1, quarter 1; q3, quarter 3.

**TABLE 3:** Top five concomitant medications for oseltamivir and baloxavir marboxil AEs from the FAERS database.

	Baloxavir marboxil, N (%)	Oseltamivir, N (%)
Concomitant Medications	Acetaminophen, 41 (26.28)	Acetaminophen, 57 (9.84)
	Carbocysteine, 21 (13.46)	Ibuprofen, 41 (7.08)
	Tipepidine hibenzate, 11 (7.05)	Ceftriaxone, 13 (2.25)
	Ambroxol, 6 (3.85)	Azithromycin, 12 (2.07)
	Dexchlorpheniramine, 4 (2.56)	Meropenem, 12 (2.07)

Abbreviations: AEs, adverse events; FAERS, united states food and drug administration adverse event reporting system; N, number of reports.

### 3.2 Signal distribution at the SOC level

**FIGURE 2:**
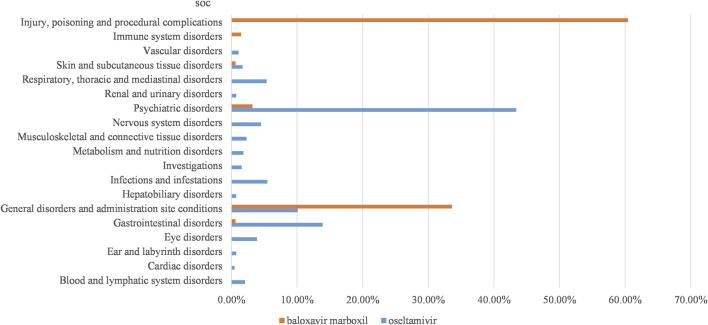
Proportion of reported cases of oseltamivir and baloxavir marboxil involving systemic AEs. Abbreviations: SOC, organ system classification.

In this study, we categorized the signaling PTs by SOC and mined out oseltamivir 100 AE signals involving 17 SOCs, baloxavir marboxil 11 AE signals involving 6 SOCs. We categorized and summarized by the cumulative number of reports for each SOC (Figure 2), which showed that the top 3 for oseltamivir were psychiatric disorders (43.41%), gastrointestinal disorders (13.90%), general disorders and administration site conditions (10.12%). The top 3 for baloxavir marboxil were injury, poisoning and procedural complications (60.44%), general disorders and administration site conditions (33.62%), psychiatric disorders (3.21%).

### 3.3 Signal at the PT level

Through data mining, we found large differences in the number and intensity of AEs and signals between oseltamivir and baloxavir marboxil at the PT level.

**FIGURE 3:**
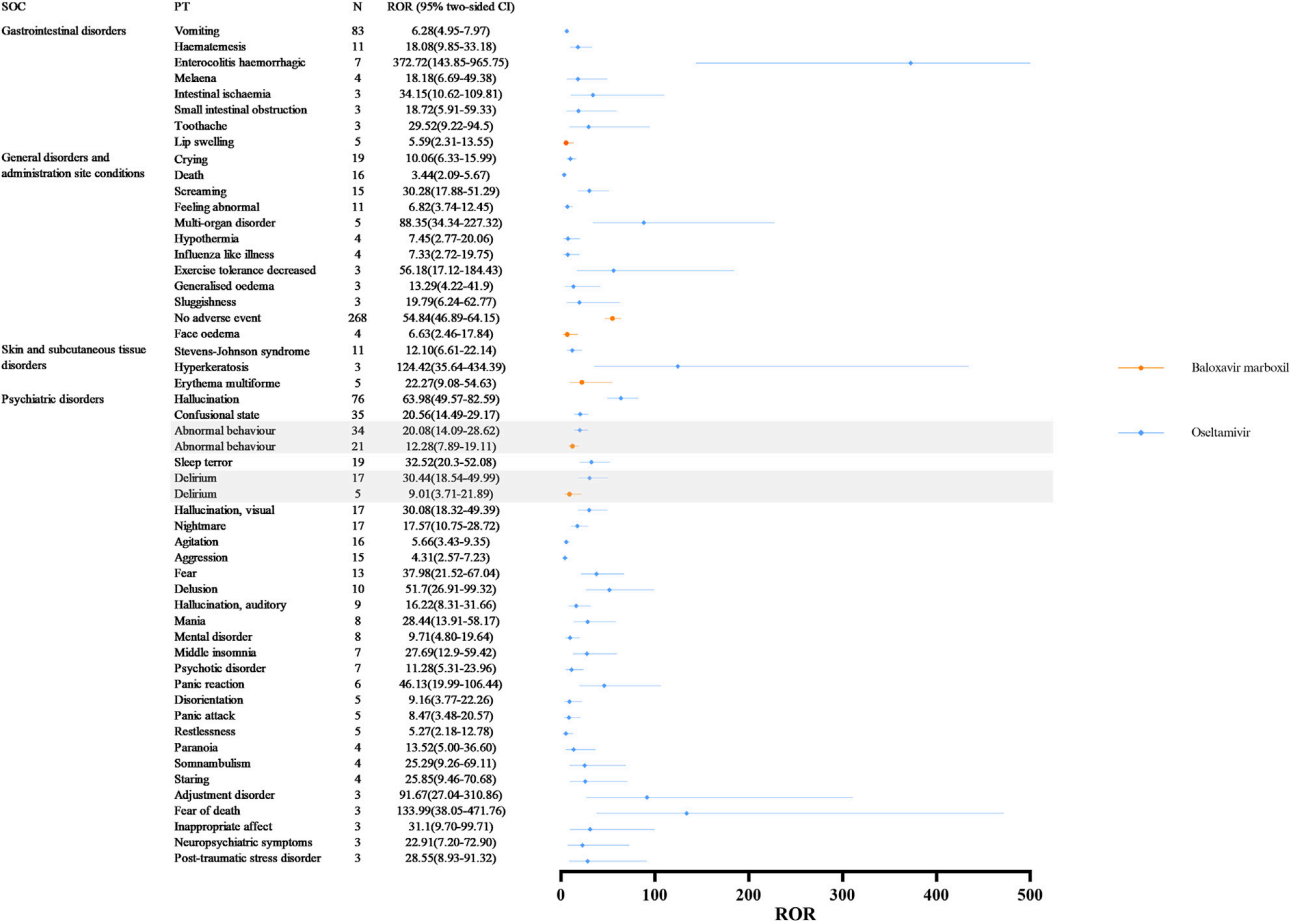
Differences in adverse event signaling in SOC Co-Involving oseltamivir and baloxavir marboxil at the PT Level. Abbreviations: SOC, organ system classification; PT, preferred term; N, number of reports; CI, Confidence Internal; ROR, reporting odds ratio.

SOCs commonly implicated in adverse event signaling for oseltamivir and baloxavir marboxil include gastrointestinal disorders, general disorders and administration site conditions, skin and subcutaneous tissue disorders, and psychiatric disorders (Figure 3 illustrates the signal strength of the ROR). Our signal mining results showed that baloxavir marboxil had a favorable safety profile, with the most reported no adverse event, with 268 cases reported, with signal intensities of ROR 54.84 (46.89–64.15), PRR 34.14 (8015.30), IC 4.97 (4.62), EBGM 31.43 (26.87). In addition, it is of interest to note that in SOC: psychiatric disorders, 21 of the children treated with baloxavir marboxil reported abnormal behavior with signal intensities of ROR 12.28 (7.89–19.11), PRR 11.75 (203.40), IC 3.53 (2.37), EBGM 11.54 (7.42). 5 cases reported delirium with signal intensities of ROR 9.01 (3.71–21.89), PRR 8.91 (34.67), IC 3.14 (0.69), EBGM 8.80 (3.62). In contrast, 34 of the children treated with oseltamivir reported abnormal behavior with signal intensities of ROR 20.08 (14.09–28.62), PRR 18.68 (553.34), IC 4.18 (3.14), EBGM 18.13 (12.72). Delirium was reported in 17 cases with signal intensity of ROR 30.44 (18.54–49.99), PRR 29.36 (443.77), IC 4.81 (2.83), and EBGM 27.99 (17.04).

Table 4 lists 5 AE signals at the PT level that were present in the two SOCs that were individually accumulated by baloxavir marboxil. Off-label use and medication errors associated with baloxavir marboxil were found to be cause for alarm in this study. Among them, 263 cases reported off label use, with signal intensities of ROR 5.60 (4.80–6.54), PRR 3.85 (610.84), IC 1.94 (1.72), and EBGM 3.82 (3.28). Intentional product use issue was reported in 203 cases with signal intensities of ROR 97.43 (81.73–116.14), PRR 66.45 (11374.74), IC 5.85 (5.28), EBGM 57.57 (48.30). Product administered to patient of inappropriate age was reported in 15 cases with signal intensities of ROR 6.16 (3.67–10.33), PRR 5.99 (62.03), IC 2.57 (1.47), EBGM 5.94 (3.54). In addition, medication error was reported in 8 cases with signal intensities of ROR 6.36 (3.15–12.84), PRR 6.26 (35.10), IC 2.63 (1.00), and EBGM 6.21 (3.07).

**TABLE 4:** The signal strength at the PT level for baloxavir marboxil-accumulated only SOC in FAERS database.

SOC	PTs	Baloxavir marboxil cases reporting PT	ROR (95% two-sided CI)	PRR (χ2)	IC (IC025)	EBGM (EBGM05)
Injury, poisoning and procedural complications	Off label use	263	5.6 (4.80–6.54)	3.85 (610.84)	1.94 (1.72)	3.82 (3.28)
	Intentional product use issue	203	97.43 (81.73–116.14)	66.45 (11374.74)	5.85 (5.28)	57.57 (48.3)
	Product administered to patient of inappropriate age	15	6.16 (3.67–10.33)	5.99 (62.03)	2.57 (1.47)	5.94 (3.54)
	Medication error	8	6.36 (3.15–12.84)	6.26 (35.10)	2.63 (1.00)	6.21 (3.07)
Anaphylactic shock	Anaphylactic shock	12	21.68 (12.10–38.84)	21.11 (222.50)	4.35 (2.25)	20.44 (11.41)

Abbreviations: PT, preferred term; SOC, system organ class; FAERS, united states food and drug administration adverse event reporting system; ROR, reporting odds ratio; CI, confidence interval; PRR, proportional reporting ratio; χ 2, chi-squared; IC, information component; EBGM, empirical Bayesian geometric mean.

The 12 SOCs that were individually accrued by oseltamivir contained 53 AE signals at the PT level (Table 5). This study first detected some signals of AEs for which the oseltamivir insert gives a warning. 11 of them reported Stevens-Johnson syndrome (SJS) with signal intensities of ROR 12.10 (6.61–22.14), PRR 11.84 (107.18), IC 3.54 (1.80), and EBGM 11.62 (6.35). In the SOC “Infections and infestations,” this study detected AE signals at the PT level, including cytomegalovirus infection reactivation, central nervous system infection, pneumonia necrotising, and pneumonia streptococcal. It is noteworthy that, in the SOC “eye disorders,” we identified 8 unexpected AE signals beyond the drug insert. Among them, 3 cases reported acute macular outer retinopathy, with a remarkably high signal strength, having values of ROR 1741.93 (180.85–16777.66), PRR 1730.67 (1296.52), IC 8.76 (0.70), and EBGM 433.42 (45.00).

**TABLE 5:** The signal strength at the PT level for oseltamivir-accumulated only SOC in FAERS database.

SOC	PTs	Oseltamivir cases reporting PT	ROR (95% two-sided CI)	PRR (χ2)	IC (IC025)	EBGM (EBGM05)
Eye disorders	Visual impairment	7	5.77 (2.72–12.21)	5.70 (26.91)	2.50 (0.79)	5.65 (2.67)
	Blindness	5	12.67 (5.20–30.87)	12.54 (52.02)	3.62 (0.85)	12.30 (5.05)
	Central vision loss	4	1163.8 (212.64–6369.79)	1153.78 (1535.74)	8.59 (1.13)	385.26 (70.39)
	Eye movement disorder	4	9.41 (3.49–25.39)	9.34 (29.35)	3.20 (0.41)	9.21 (3.41)
	Acute macular outer retinopathy	3	1741.93 (180.85–16777.66)	1730.67 (1296.52)	8.76 (0.70)	433.42 (45.00)
	Blindness unilateral	3	37.86 (11.73–122.17)	37.62 (100.42)	5.14 (0.30)	35.38 (10.96)
	Conjunctival haemorrhage	3	42.48 (13.11–137.67)	42.21 (112.49)	5.30 (0.31)	39.4 (12.16)
	Symblepharon	3	348.38 (83.01–1462.04)	346.13 (645.28)	7.76 (0.53)	216.71 (51.64)
Blood and lymphatic system disorders	Leukopenia	10	7.99 (4.25–15.01)	7.84 (59.02)	2.95 (1.39)	7.75 (4.12)
	Normocytic anaemia	4	166.25 (54.52–506.98)	164.83 (506.62)	7.00 (0.96)	128.42 (42.11)
	Lymphocytosis	3	42.48 (13.11–137.67)	42.21 (112.49)	5.30 (0.31)	39.40 (12.16)
Vascular disorders	Shock	5	5.61 (2.31–13.59)	5.56 (18.55)	2.46 (0.41)	5.51 (2.27)
	Shock haemorrhagic	4	31.45 (11.45–86.36)	31.18 (110.9)	4.89 (0.76)	29.64 (10.79)
Cardiac disorders	Supraventricular tachycardia	4	13.6 (5.03–36.82)	13.49 (45.25)	3.72 (0.55)	13.21 (4.88)
Renal and urinary disorders	Incontinence	3	42.48 (13.11–137.67)	42.21 (112.49)	5.30 (0.31)	39.4 (12.16)
	Kidney enlargement	3	48.38 (14.85–157.66)	48.07 (127.67)	5.47 (0.33)	44.45 (13.64)
Respiratory, thoracic and mediastinal disorders	Acute respiratory distress syndrome	7	7.82 (3.69–16.59)	7.72 (40.49)	2.93 (1.02)	7.63 (3.60)
	Pleural effusion	6	5.77 (2.57–12.97)	5.71 (23.15)	2.50 (0.63)	5.67 (2.52)
	Lung disorder	5	7.87 (3.24–19.11)	7.80 (29.27)	2.95 (0.62)	7.71 (3.17)
	Obliterative bronchiolitis	5	45.55 (18.25–113.70)	45.07 (199.90)	5.39 (1.20)	41.88 (16.78)
	Aphonia	3	60.06 (18.23–197.86)	59.68 (156.88)	5.76 (0.35)	54.18 (16.45)
	Haemothorax	3	66.99 (20.21–222.11)	66.56 (173.71)	5.90 (0.36)	59.78 (18.03)
	Hypercapnia	3	20.98 (6.61–66.63)	20.85 (54.74)	4.33 (0.20)	20.16 (6.35)
	Obstructive airways disorder	3	10.75 (3.42–33.79)	10.68 (25.87)	3.39 (0.03)	10.51 (3.34)
	Oropharyngeal oedema	3	96.77 (28.41–329.64)	96.15 (242.14)	6.37 (0.40)	82.56 (24.23)
	Pulmonary fibrosis	3	37.06 (11.49–119.48)	36.82 (98.30)	5.12 (0.29)	34.67 (10.75)
	Pulmonary necrosis	3	870.96 (145.19–5224.71)	865.34 (1036.02)	8.44 (0.63)	346.73 (57.8)
Musculoskeletal and connective tissue disorders	Muscular weakness	6	5.84 (2.60–13.12)	5.78 (23.53)	2.52 (0.64)	5.73 (2.55)
	Musculoskeletal stiffness	4	6.46 (2.40–17.37)	6.41 (18.09)	2.67 (0.22)	6.35 (2.36)
	Compartment syndrome	3	28.09 (8.79–89.81)	27.91 (74.27)	4.74 (0.25)	26.67 (8.34)
	Muscle rigidity	3	10.06 (3.20–31.62)	10.00 (23.91)	3.30 (0.01)	9.85 (3.13)
	Osteopenia	3	16.27 (5.15–51.45)	16.17 (41.56)	3.98 (0.14)	15.76 (4.99)
Nervous system disorders	Tremor	12	4.33 (2.43–7.69)	4.24 (29.67)	2.08 (0.96)	4.22 (2.37)
	Loss of consciousness	9	4.38 (2.26–8.49)	4.31 (22.83)	2.10 (0.77)	4.29 (2.21)
	Incoherent	4	42.31 (15.27–117.24)	41.96 (149.11)	5.29 (0.81)	39.18 (14.14)
	Dysgraphia	3	28.09 (8.79–89.81)	27.91 (74.27)	4.74 (0.25)	26.67 (8.34)
	Muscle contractions involuntary	3	39.58 (12.25–127.93)	39.33 (104.94)	5.21 (0.30)	36.89 (11.41)
	Neuralgia	3	14.63 (4.64–46.18)	14.54 (36.92)	3.83 (0.12)	14.21 (4.50)
	Slow response to stimuli	3	40.5 (12.52–131.02)	40.25 (107.35)	5.24 (0.30)	37.69 (11.65)
Investigations	Body temperature decreased	6	29.7 (13.01–67.81)	29.33 (156.33)	4.81 (1.41)	27.96 (12.25)
	Influenza A virus test positive	4	211.59 (67.13–666.95)	209.78 (609.53)	7.27 (0.98)	154.1 (48.89)
	Procalcitonin increased	3	35.54 (11.04–114.44)	35.32 (94.29)	5.06 (0.29)	33.34 (10.35)
Infections and infestations	Pneumonia	18	3.45 (2.15–5.53)	3.35 (29.86)	1.74 (0.92)	3.34 (2.08)
	Influenza	14	5.33 (3.12–9.10)	5.20 (47.35)	2.37 (1.28)	5.16 (3.03)
	Cytomegalovirus infection reactivation	4	6.53 (2.43–17.57)	6.48 (18.36)	2.68 (0.23)	6.42 (2.39)
	Central nervous system infection	3	79.17 (23.61–265.44)	78.67 (202.45)	6.12 (0.38)	69.35 (20.68)
	Pneumonia necrotising	3	54.43 (16.61–178.37)	54.08 (142.93)	5.63 (0.34)	49.53 (15.11)
	Pneumonia streptococcal	3	60.06 (18.23–197.86)	59.68 (156.88)	5.76 (0.35)	54.18 (16.45)
Hepatobiliary disorders	Hepatitis cholestatic	3	23.53 (7.39–74.91)	23.39 (61.80)	4.49 (0.22)	22.52 (7.07)
	Hepatosplenomegaly	3	10.81 (3.44–34.00)	10.75 (26.06)	3.40 (0.03)	10.57 (3.36)
Ear and labyrinth disorders	Ear disorder	3	32.25 (10.05–103.52)	32.05 (85.51)	4.93 (0.27)	30.42 (9.48)
	Ear inflammation	3	435.48 (97.19–1951.2)	432.67 (738.31)	7.95 (0.56)	247.67 (55.28)
Metabolism and nutrition disorders	Dehydration	15	7.79 (4.64–13.07)	7.57 (84.80)	2.90 (1.71)	7.49 (4.46)

Abbreviations: PT, preferred term; SOC, system organ class; FAERS, united states food and drug administration adverse event reporting system; ROR, reporting odds ratio; CI, confidence interval; PRR, proportional reporting ratio; χ 2, chi-squared; IC, information component; EBGM, empirical Bayesian geometric mean.

### 3.4 Subgroup analysis by gender

**FIGURE 4:**
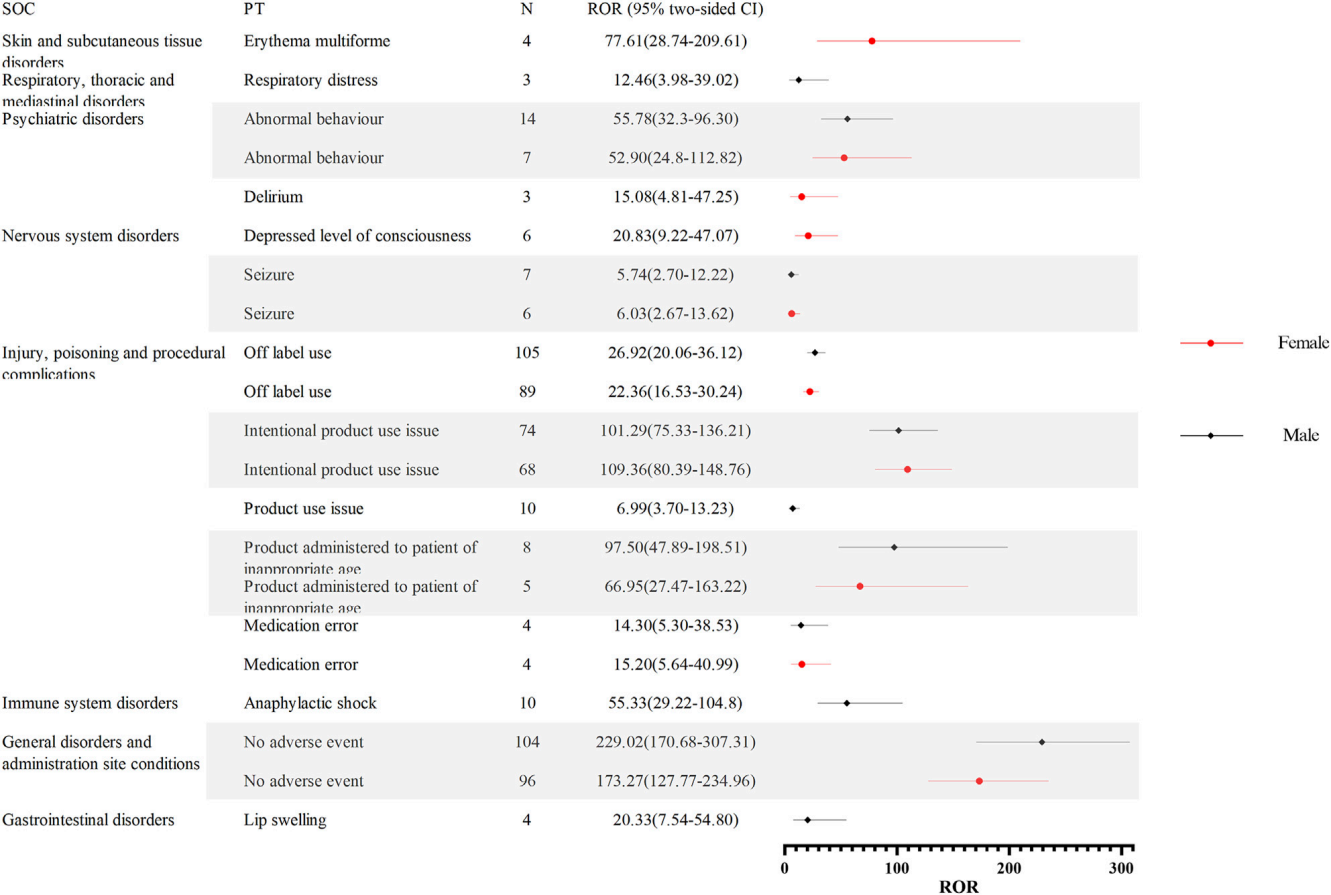
Distribution of AE signal intensities for baloxavir marboxil in male and female children based on ROR calculation method. Abbreviations: AE, adverse event; ROR, reporting odds ratio; SOC, organ system classification; PT, preferred term; N, number of reports; CI, Confidence Internal.

**FIGURE 5:**
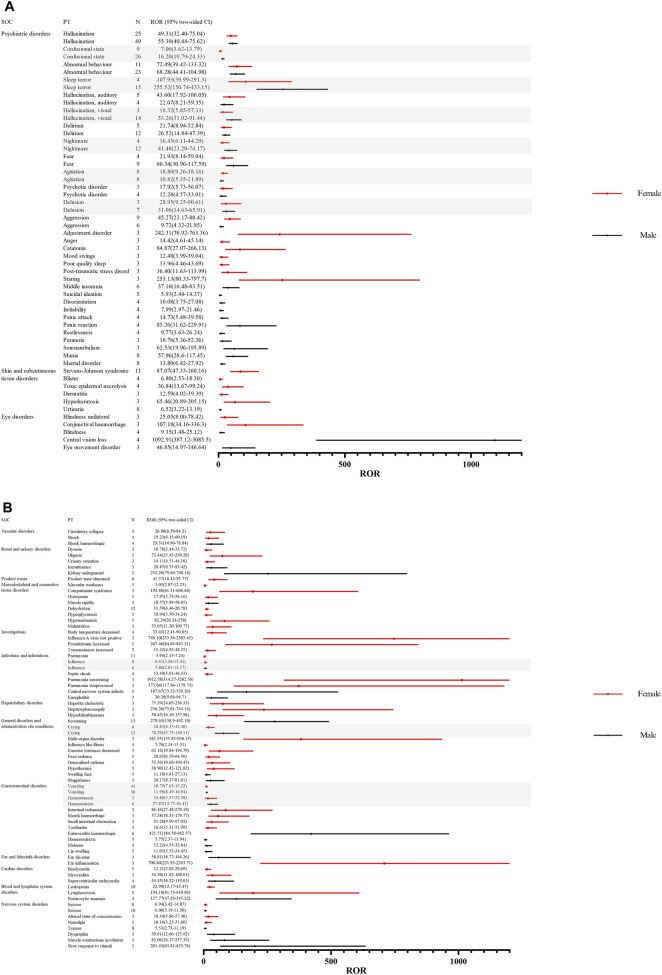
**(A)**. Distribution of AE signal intensities for oseltamivir in male and female children based on ROR calculation method. Abbreviations: AE, adverse event; ROR, reporting odds ratio; SOC, organ system classification; PT, preferred term; N, number of reports; CI, Confidence Internal. **(B)**. Distribution of AE signal intensities for oseltamivir in male and female children based on ROR calculation method .

To further investigate the differences in adverse event signaling between male and female children, we stratified the analysis by gender for children receiving oseltamivir and baloxavir, respectively. Figure 4 demonstrates the distribution of AE signal intensities based on the ROR calculation method for baloxavir marboxil. Overall, the signal of AEs due to baloxavir marboxil was approximately the same in male and female pediatric patients. However, it is of concern that anaphylactic shock was reported in 10 male children with signal intensities of ROR 55.33 (29.22–104.80), PRR 52.35 (502.33), IC 5.70 (2.34), and EBGM 52.16 (27.54). In contrast, in female children no such AE signal was found. For oseltamivir, Figures 5A,B show the distribution of AE signals between genders. Overall, there were more AE signals in girls than boys. Alarmingly, in skin and subcutaneous tissue disorders, SJS was reported in 11 girls with signal intensities of ROR 87.07 (47.33–160.16), PRR 82.26 (879.74), IC 6.36 (2.59), and EBGM 81.91 (44.53). In addition, four girls reported toxic epidermal necrolysis (TEN) with the signal intensity of ROR 36.84 (13.67–99.24), PRR 36.11 (136.36), IC 5.17 (0.78), and EBGM 36.04 (13.38). Supplementary Table 1 through 4 display the results of the four signal calculation methods.

## 4 Discussion

This is the first and most thorough post-marketing pharmacovigilance study of oseltamivir and baloxavir marboxil in children using the FAERS database. Our study reveals real-world differences in AEs that occur in children treated with oseltamivir and baloxavir marboxil.

Baloxavir inhibits the endonuclease activity of the polymerase acidic (PA) protein, an influenza virus-specific enzyme in the viral RNA polymerase complex required for viral gene transcription, resulting in inhibition of influenza virus replication (Heo, 2018). Baloxavir marboxil is effective against oseltamivir-resistant influenza viruses and may act synergistically with neuraminidase inhibitors. Oseltamivir has a half-life of 1–3 h. The half-life of baloxavir can reach 79 h (Table 6 summarizes the pharmacokinetic (PK) properties of baloxavir and oseltamivir) (Abraham et al., 2020). This implies that baloxavir marboxil only requires a single dose. Among children treated with baloxavir marboxil, we detected an AE signal for medication error. Medication errors are a major cause of harm in the healthcare system and can be prevented (Mirosevic Skvrce et al., 2020). Medication errors are one of the most common causes of pediatric AEs. They are more likely to occur in pediatric patients than adults, and dosing errors are the most common cause (Mirosevic Skvrce et al., 2024). Previous studies have pointed out that the PT “no adverse event” was frequently recorded in children and adolescent age groups along with medication error PTs with signals of disproportionate reporting, and pediatric patients are more vulnerable to the harm caused by medication errors (Carnovale et al., 2018), which is consistent with our findings. For children with influenza treated with baloxavir marboxil, the drug needs to be administered only once during the entire course of the illness. This is compared to traditional dosing methods (e.g., oseltamivir needs to be given twice daily for 5 days). Baloxavir marboxil‘s unique dosing frequency undoubtedly increases the risk of medication errors. Therefore, medication education for children and their families is essential.

**TABLE 6:** Comparison of baloxavir marboxil and oseltamivir Mechanism of Action, Microbiology, and Pharmacokinetics.

	Baloxavir marboxil	Oseltamivir
Mechanism of action	Endonuclease inhibitor	Neuraminidase inhibitor
Microbiology	Influenza type A and B viruses, including NAI-resistant strains	Influenza type A and B viruses
Protein-binding, %	>90	42
Volume of distribution, L	1180	23–26
Half-life, hours	79	1–3

Abbreviation: NAI, neuraminidase inhibitor.

In addition, there was an AE signal for off-label use in children treated with baloxavir marboxil. Off-label use is common and usually legal, unless it violates ethical guidelines or safety regulations. The reason for this is usually to fulfill a patient’s medical need or to make an innovative drug available to the patient, especially if there are no other better options available (Wu and Wu, 2014; Rusz et al., 2021). Far fewer drugs are approved for use in children than are approved for use in adults. Therefore, off-label drug use in children is more common. Moreover, off-label drug use in children typically lacks the same level of evidence as off-label use authorized for adults (Kaley et al., 2019). The minimum age for approved use of baloxavir varies in different countries, with the European Union recommending it for patients aged 1 year and older with influenza, and Japan allowing it to be used in children aged <12 years and weighing ≥10 kg with influenza. However, currently, the FDA and countries like China only approve the use of baloxavir marboxil in children aged 5 and above. For oseltamivir, the age range for its use is wider, as the FDA approved it for use in children ≥3 months of age. For children under 5 years old with influenza virus infections resistant to neuraminidase inhibitors, baloxavir marboxil may be a reliable alternative. Consequently, the issue of off-label use is inevitably raised. If the benefits outweigh the risks upon evaluation, reasonable and clinically appropriate off-label prescriptions for children should be considered (Schrier et al., 2020; van der Zanden et al., 2021).

In our study of oseltamivir, a number of AE signals consistent with warnings and precautions in the drug’s labeling were identified, including severe skin/allergic reactions, neuropsychiatric events, and bacterial infections, which confirms the reliability of our findings. However, of the 8 AE signals in SOC: eye disorders not mentioned in the oseltamivir instructions, acute macular outer retinopathy is particularly alarming. Acute macular neuroretinopathy (AMN) is a rare retinal disorder characterized by acute, symptomatic photopsias and paracentral scotomas associated with mild vision loss (Bos and Deutman, 1975). It has now been shown that acute influenza virus circulating cytokine levels are elevated during acute influenza virus infection (Bian et al., 2014), and elevated levels of tumor necrosis factor alpha (TNFα) may damage retinal photoreceptors *in vitro* and lead to mitochondrial dysfunction and visual datasource cell death in models of ischemic brain injury (Nakazawa et al., 2011; Doll et al., 2015). Another study also noted that approximately 50% of the 101 cases of AMN were associated with prior influenza illness (Bhavsar et al., 2016). However, to date, there has been no robust evidence linking AMN to influenza. Given the exploratory nature of our study, the detection of a signal for acute macular outer retinopathy warrants further investigation. While we identified this potential association, it is important to interpret these findings with caution. Additional research is needed to confirm any causal relationship and to understand the underlying mechanisms.

The field of medicine is gradually recognizing the impact of gender on treatment outcomes, and women are increasingly recognized as risk factors for developing side effects with significant consequences (Franconi and Campesi, 2014). Furthermore, research indicates that age and gender influence the frequency of unprompted complaints about adverse reactions (Holm et al., 2017). As a result, in this study, we investigated the use of oseltamivir and baloxavir marboxil with real-world AE signals. Based on the study’s findings, we observed signals of AEs caused by oseltamivir only in female patients: SJS and TEN. SJS/TEN is a severe skin-mucosal reaction, caused mainly by drugs, characterized by blistering and generalized epidermolysis bullosa (White et al., 2018). In the United States, the estimated incidence of SJS, overlapping SJS/TEN, and TEN in children was 5.3 cases per 1 million, 0.8 cases per 1 million, and 0.4 cases per 1 million, respectively (Hsu et al., 2017). In addition, the results of one study indicate that SJS/TEN is more prevalent in women, with a male-to-female ratio of approximately 1:2 (Sekula et al., 2013). Therefore, healthcare professionals and patients’ families should be more alert to the occurrence of such cutaneous severe AEs in female children treated with oseltamivir. As for children treated with baloxavir, it is of concern that the AE signal for anaphylactic shock was only seen in male patients. Anaphylactic shock is a fatal allergic reaction (Li et al., 2022). Compared to adult data, reports of drug-induced anaphylactic reactions (DIA) in children are rare (Bianchi et al., 2024). DIA is primarily an IgE-mediated tachyphylactic reaction leading to the degranulation of mast cells and basophils and the release of pro-inflammatory mediators (Pichler, 2003). The pathogens that cause drug-induced systemic anaphylactic reactions may be different depending on the country and the way the data is collected. Antibiotics (mainly penicillins and cephalosporins) are usually the drugs most often linked to fatal drug-induced anaphylactic reactions. Still, other drugs like neuromuscular blocking agents and radiographic contrast agents have also been linked (Turner et al., 2017). In a French study of severe allergies associated with neuromuscular blockers, men were a risk factor associated with fatal outcomes (Reitter et al., 2014). Although the mechanisms involved have not been elucidated, our study still reveals a high association with anaphylactic shock in male children treated with baloxavir marboxil, which emphasizes the importance of close monitoring and follow-up of this severe allergic reaction, especially in male children treated with baloxavir marboxil.

Combination therapy is often considered a promising strategy to circumvent treatment resistance. When used together, drugs that work against different viral proteins or host factors may help stop the development of resistant strains even more than when they are used alone (F. Hayden, 2009; Govorkova and Webster, 2010). Preclinical studies have shown that nizoralnit or itraconazole combined with oseltamivir can be more effective at killing viruses than oseltamivir alone (Belardo et al., 2015; Schloer et al., 2020). As for baloxavir, the results of the current preclinical study showed that combination therapy with oseltamivir or famciclovir increased *in vitro* antiviral activity (Checkmahomed et al., 2020), combination therapy with influenza drugs with different mechanisms of action decreased the selection pressure for viruses with reduced drug susceptibility (Hamza et al., 2021; Park et al., 2021; Koszalka et al., 2022). However, many challenges remain in managing the risk of drug interactions in clinical practice. Combination drug interactions may occur at the PK level, where a drug can affect the absorption, metabolism, elimination, induction, or inhibition of other drug metabolizing enzymes. This may result in changes in drug concentration, leading to toxicity or reduced efficacy (van Hasselt and Iyengar, 2019). An RCT involving 366 patients demonstrated a well-tolerated combination of baloxavir marboxil and NAIs compared to NAIs alone, with no new safety signals observed (Kumar et al., 2022). In the present study, only three cases of AE with baloxavir marboxil as the PS drug and oseltamivir as a co-administration and two cases of AE with oseltamivir as the PS drug and baloxavir marboxil as a co-administration were reported (Supplementary Tables S5, S6). Therefore, we were unable to assess whether the combination of the two would increase the incidence of adverse events or lead to new AE signals. More high-quality studies are required in the future to examine the safety of oseltamivir and baloxavir marboxil.

We analyzed AE signals linked to oseltamivir and baloxavir marboxil using the FAERS database for disproportionate analysis. This approach displays strong extrapolation skills, successfully overcoming the constraints of small sample numbers and short observation periods in clinical studies. However, it is crucial to recognize and address some limitations. First, FAERS is a spontaneous reporting system that gathers data from many nations and professions. It may include missing or inaccurate information, which might introduce bias in the study. Second, the lack of the total number of pediatric patients treated with oseltamivir or baloxavir marboxil made it impossible to calculate the incidence of each AE. Third, this study did not establish a direct cause-and-effect relationship between drugs and AEs. Disproportionate analyses only indicate the strength of a signal statistically, without quantifying risk or causality. Further research is required to confirm the experimental findings. Finally, although we performed data cleaning and de-duplication operations as recommended by the FDA, we may still retain potential duplicate entries that may exaggerate the strength of certain AE signals (Schilder et al., 2023; Cutroneo et al., 2024). Despite these limitations, our findings offer valuable insights for healthcare providers and patients regarding the monitoring of AEs associated with oseltamivir and baloxavir marboxil in children.

## 5 Conclusion

This pharmacovigilance study explored reports of AEs associated with oseltamivir and baloxavir marboxil use in children in the FAERS database. This long-term post-marketing drug safety evaluation provides an overview of the safety profiles of oseltamivir and baloxavir marboxil in children. Our exploratory findings suggest an AE signal of acute macular outer retinopathy in children treated with oseltamivir, indicating a need for further investigation to confirm or reject this association. For children treated with baloxavir, the frequency of administration poses a risk of medication errors, highlighting the importance of proper medication education.

## Data Availability

The raw data supporting the conclusions of this article will be made available by the authors, without undue reservation.
