# Supplementary material for: The real-world safety of oseltamivir and baloxavir marboxil in children: a disproportionality analysis of the FDA adverse event reporting system
Source: Front Pharmacol. 2024 Jul 10;15:1391003. doi: 10.3389/fphar.2024.1391003 (PMC11266138; doi:10.3389/fphar.2024.1391003)
Supplement: Supplementary file 1 [file Table1.DOCX]

Supplementary Table 1. Signal intensity of baloxavir marboxil reported at the preferred term (PT) level in male pediatric patients.

| SOC | Preferred terms (PTs) | baloxavir marboxil cases reporting PT | ROR (95%two-  sided CI) | PRR(χ2) | IC  (IC025) | EBGM  (EBGM05) |
| --- | --- | --- | --- | --- | --- | --- |
| Respiratory, thoracic and mediastinal disorders | Respiratory distress | 3 | 12.46(3.98-39.02) | 12.27(31.07) | 3.62(0.07) | 12.26(3.92) |
| Psychiatric disorders | Abnormal behaviour | 14 | 55.78(32.30-96.30) | 51.56(692.64) | 5.68(2.84) | 51.38(29.76) |
| Nervous system disorders | Seizure | 7 | 5.74(2.70-12.22) | 5.56(26.33) | 2.47(0.76) | 5.55(2.61) |
| Injury, poisoning and procedural complications | Off label use | 105 | 26.92(20.06-36.12) | 11.96(1107.58) | 3.58(3.09) | 11.95(8.91) |
|  | Intentional product use issue | 74 | 101.29(75.33-136.21) | 60.51(4341.91) | 5.91(4.72) | 60.26(44.81) |
|  | Product use issue | 10 | 6.99(3.70-13.23) | 6.66(48.51) | 2.74(1.25) | 6.66(3.52) |
|  | Product administered to patient of inappropriate age | 8 | 97.50(47.89-198.51) | 93.26(725.66) | 6.53(2.08) | 92.65(45.51) |
|  | Medication error | 4 | 14.30(5.30-38.53) | 14.01(48.33) | 3.81(0.56) | 13.99(5.19) |
| Immune system disorders | Anaphylactic shock | 10 | 55.33(29.22-104.8) | 52.35(502.33) | 5.70(2.34) | 52.16(27.54) |
| General disorders and administration site conditions | No adverse event | 104 | 229.02(170.68-307.31) | 98.72(10048.46) | 6.62(5.37) | 98.04(73.06) |
| Gastrointestinal disorders | Lip swelling | 4 | 20.33(7.54-54.80) | 19.90(71.79) | 4.31(0.66) | 19.88(7.37) |

Supplementary Table 2. Signal intensity of baloxavir marboxil reported at the preferred term (PT) level in female pediatric patients.

| SOC | Preferred terms (PTs) | baloxavir marboxil cases reporting PT | ROR (95%two-  sided CI) | PRR(χ2) | IC  (IC025) | EBGM  (EBGM05) |
| --- | --- | --- | --- | --- | --- | --- |
| Skin and subcutaneous tissue disorders | Erythema multiforme | 4 | 77.61(28.74-209.61) | 75.80(294.32) | 6.24(0.86) | 75.54(27.97) |
| Psychiatric disorders | Abnormal behaviour | 7 | 52.90(24.8-112.82) | 50.75(340.85) | 5.66(1.77) | 50.63(23.74) |
|  | Delirium | 3 | 15.08(4.81-47.25) | 14.83(38.71) | 3.89(0.12) | 14.82(4.73) |
| Nervous system disorders | Depressed level of consciousness | 6 | 20.83(9.22-47.07) | 20.13(109.16) | 4.33(1.30) | 20.11(8.90) |
|  | Seizure | 6 | 6.03(2.67-13.62) | 5.85(24.28) | 2.55(0.64) | 5.85(2.59) |
| Injury, poisoning and procedural complications | Off label use | 89 | 22.36(16.53-30.24) | 11.11(859.07) | 3.47(2.95) | 11.10(8.21) |
|  | Intentional product use issue | 68 | 109.36(80.39-148.76) | 65.76(4350.13) | 6.03(4.72) | 65.56(48.19) |
|  | Product administered to patient of inappropriate age | 5 | 66.95(27.47-163.22) | 65.00(314.29) | 6.02(1.24) | 64.81(26.59) |
|  | Medication error | 4 | 15.20(5.64-40.99) | 14.86(51.76) | 3.89(0.58) | 14.85(5.51) |
| General disorders and administration site conditions | No adverse event | 96 | 173.27(127.77-234.96) | 75.41(7077.83) | 6.23(5.09) | 75.15(55.42) |

ROR, reporting odds ratio; CI, confidence interval; PRR, proportional reporting ratio; χ 2, chi-squared; IC, information component; EBGM, empirical Bayesian geometric mean; SOC, system organ class.

Supplementary Table 3. Signal intensity of oseltamivir reported at the preferred term (PT) level in male pediatric patients.

| SOC | Preferred terms (PTs) | baloxavir marboxil cases reporting PT | ROR (95%two-  sided CI) | PRR(χ2) | IC  (IC025) | EBGM  (EBGM05) |
| --- | --- | --- | --- | --- | --- | --- |
| Vascular disorders | Shock haemorrhagic | 4 | 29.31(10.9-78.84) | 28.86(107.33) | 4.85(0.74) | 28.78(10.7) |
| Skin and subcutaneous tissue disorders | Urticaria | 8 | 6.52(3.22-13.19) | 6.34(36.17) | 2.66(1.01) | 6.34(3.13) |
| Renal and urinary disorders | Incontinence | 3 | 29.87(9.55-93.42) | 29.53(82.47) | 4.88(0.25) | 29.44(9.42) |
|  | Kidney enlargement | 3 | 252.2(79.69-798.18) | 249.17(723.9) | 7.93(0.38) | 243.26(76.86) |
| Psychiatric disorders | Hallucination | 49 | 55.3(40.44-75.62) | 44.61(2089.22) | 5.47(4.17) | 44.42(32.48) |
|  | Confusional state | 26 | 16.2(10.79-24.33) | 14.62(331.7) | 3.87(2.73) | 14.6(9.72) |
|  | Abnormal behaviour | 23 | 68.28(44.41-104.98) | 62.06(1375.52) | 5.95(3.57) | 61.69(40.13) |
|  | Sleep terror | 15 | 255.52(150.74-433.15) | 240.19(3491.7) | 7.87(3.22) | 234.69(138.45) |
|  | Hallucination, visual | 14 | 53.26(31.02-91.44) | 50.33(674.27) | 5.65(2.83) | 50.08(29.17) |
|  | Delirium | 12 | 26.52(14.84-47.39) | 25.29(279.76) | 4.66(2.35) | 25.23(14.12) |
|  | Nightmare | 12 | 41.48(23.2-74.17) | 39.53(449.51) | 5.3(2.53) | 39.38(22.03) |
|  | Fear | 9 | 60.34(30.96-117.59) | 58.19(503.33) | 5.85(2.2) | 57.87(29.69) |
|  | Agitation | 8 | 10.82(5.35-21.89) | 10.5(68.94) | 3.39(1.37) | 10.5(5.19) |
|  | Mania | 8 | 57.96(28.6-117.45) | 56.13(431.08) | 5.8(2.01) | 55.83(27.55) |
|  | Mental disorder | 8 | 13.8(6.82-27.92) | 13.39(91.79) | 3.74(1.52) | 13.37(6.61) |
|  | Delusion | 7 | 31.06(14.63-65.91) | 30.21(197.32) | 4.91(1.66) | 30.13(14.19) |
|  | Aggression | 6 | 9.72(4.32-21.85) | 9.51(45.74) | 3.25(0.96) | 9.5(4.22) |
|  | Middle insomnia | 6 | 37.1(16.48-83.51) | 36.23(204.94) | 5.17(1.46) | 36.1(16.04) |
|  | Suicidal ideation | 5 | 5.93(2.44-14.37) | 5.83(20.06) | 2.54(0.44) | 5.83(2.4) |
|  | Disorientation | 4 | 10.08(3.75-27.08) | 9.93(32.15) | 3.31(0.44) | 9.92(3.69) |
|  | Hallucination, auditory | 4 | 22.07(8.21-59.35) | 21.74(79.02) | 4.44(0.68) | 21.69(8.07) |
|  | Irritability | 4 | 7.99(2.97-21.46) | 7.87(24.03) | 2.98(0.33) | 7.87(2.93) |
|  | Panic attack | 4 | 14.73(5.48-39.58) | 14.51(50.28) | 3.86(0.57) | 14.49(5.39) |
|  | Panic reaction | 4 | 85.26(31.62-229.91) | 83.91(325.08) | 6.38(0.87) | 83.23(30.87) |
|  | Psychotic disorder | 4 | 12.28(4.57-33.01) | 12.1(40.74) | 3.6(0.51) | 12.09(4.5) |
|  | Restlessness | 4 | 9.77(3.63-26.24) | 9.63(30.94) | 3.27(0.42) | 9.62(3.58) |
|  | Paranoia | 3 | 16.76(5.36-52.36) | 16.57(43.84) | 4.05(0.15) | 16.54(5.29) |
|  | Somnambulism | 3 | 62.53(19.96-195.89) | 61.79(178.38) | 5.94(0.32) | 61.42(19.61) |
| Nervous system disorders | Seizure | 10 | 6(3.19-11.3) | 5.8(40.01) | 2.54(1.13) | 5.8(3.08) |
|  | Tremor | 8 | 5.53(2.73-11.19) | 5.39(28.73) | 2.43(0.87) | 5.38(2.66) |
|  | Dysgraphia | 3 | 39.61(12.66-123.92) | 39.14(111.11) | 5.29(0.28) | 39(12.46) |
|  | Muscle contractions involuntary | 3 | 82.06(26.17-257.33) | 81.08(235.44) | 6.33(0.34) | 80.45(25.65) |
|  | Slow response to stimuli | 3 | 201.43(63.82-635.76) | 199.01(579.79) | 7.61(0.38) | 195.23(61.85) |
| Musculoskeletal and connective tissue disorders | Muscle rigidity | 3 | 18.57(5.94-58.05) | 18.36(49.2) | 4.2(0.17) | 18.33(5.87) |
| Infections and infestations | Influenza | 6 | 5.86(2.61-13.17) | 5.74(23.58) | 2.52(0.63) | 5.74(2.55) |
|  | Central nervous system infection | 3 | 167.67(53.22-528.26) | 165.66(483.21) | 7.35(0.37) | 163.04(51.75) |
|  | Encephalitis | 3 | 30.28(9.68-94.7) | 29.93(83.68) | 4.9(0.25) | 29.85(9.54) |
| General disorders and administration site conditions | Screaming | 13 | 279.63(158.9-492.1) | 265.09(3334.33) | 8.01(2.99) | 258.41(146.84) |
|  | Crying | 12 | 78.29(43.75-140.11) | 74.57(865.24) | 6.21(2.7) | 74.04(41.37) |
|  | Swelling face | 5 | 11.18(4.61-27.13) | 10.98(45.39) | 3.46(0.8) | 10.97(4.52) |
|  | Sluggishness | 3 | 26.17(8.37-81.81) | 25.86(71.56) | 4.69(0.23) | 25.8(8.25) |
| Gastrointestinal disorders | Vomiting | 38 | 11.99(8.49-16.94) | 10.31(324.1) | 3.37(2.59) | 10.3(7.29) |
|  | Haematemesis | 8 | 27.87(13.77-56.42) | 27.01(200.07) | 4.75(1.82) | 26.94(13.31) |
|  | Enterocolitis haemorrhagic | 6 | 421.51(184.58-962.57) | 411.38(2361.37) | 8.63(1.67) | 395.5(173.19) |
|  | Haematochezia | 5 | 5.75(2.37-13.94) | 5.66(19.22) | 2.5(0.42) | 5.65(2.33) |
|  | Melaena | 4 | 12.22(4.55-32.84) | 12.04(40.5) | 3.59(0.51) | 12.03(4.48) |
|  | Lip swelling | 3 | 11.03(3.53-34.45) | 10.91(27) | 3.45(0.03) | 10.9(3.49) |
| Eye disorders | Blindness | 4 | 9.35(3.48-25.12) | 9.22(29.32) | 3.2(0.4) | 9.21(3.43) |
|  | Central vision loss | 4 | 1092.91(387.12-3085.5) | 1075.37(3884.63) | 9.93(0.96) | 973.05(344.66) |
|  | Eye movement disorder | 3 | 46.85(14.97-146.64) | 46.3(132.39) | 5.53(0.3) | 46.09(14.73) |
| Cardiac disorders | Supraventricular tachycardia | 4 | 44.45(16.52-119.63) | 43.75(166.45) | 5.45(0.8) | 43.57(16.19) |
| Blood and lymphatic system disorders | Normocytic anaemia | 4 | 127.77(47.29-345.22) | 125.74(489.02) | 6.96(0.89) | 124.22(45.98) |

ROR, reporting odds ratio; CI, confidence interval; PRR, proportional reporting ratio; χ 2, chi-squared; IC, information component; EBGM, empirical Bayesian geometric mean; SOC, system organ class.

Supplementary Table 4. Signal intensity of oseltamivir reported at the preferred term (PT) level in female pediatric patients.

| SOC | Preferred terms (PTs) | baloxavir marboxil cases reporting PT | ROR (95%two-  sided CI) | PRR(χ2) | IC  (IC025) | EBGM  (EBGM05) |
| --- | --- | --- | --- | --- | --- | --- |
| Vascular disorders | Circulatory collapse | 3 | 26.9(8.59-84.2) | 26.5(73.56) | 4.73(0.23) | 26.47(8.46) |
|  | Shock | 3 | 19.23(6.15-60.19) | 18.96(51.01) | 4.24(0.18) | 18.94(6.05) |
| Skin and subcutaneous tissue disorders | Stevens-Johnson syndrome | 11 | 87.07(47.33-160.16) | 82.26(879.74) | 6.36(2.59) | 81.91(44.53) |
|  | Blister | 4 | 6.8(2.53-18.3) | 6.68(19.38) | 2.74(0.24) | 6.68(2.48) |
|  | Toxic epidermal necrolysis | 4 | 36.84(13.67-99.24) | 36.11(136.36) | 5.17(0.78) | 36.04(13.38) |
|  | Dermatitis | 3 | 12.59(4.02-39.39) | 12.41(31.5) | 3.63(0.07) | 12.4(3.96) |
|  | Hyperkeratosis | 3 | 65.46(20.89-205.15) | 64.48(186.89) | 6.01(0.33) | 64.26(20.51) |
| Respiratory, thoracic and mediastinal disorders | Respiratory failure | 7 | 14.71(6.92-31.29) | 14.23(86.22) | 3.83(1.37) | 14.22(6.68) |
|  | Acute respiratory distress syndrome | 6 | 52.09(23.09-117.53) | 50.54(290.72) | 5.66(1.52) | 50.4(22.34) |
|  | Hypoxia | 6 | 21.1(9.36-47.58) | 20.49(111.27) | 4.36(1.3) | 20.47(9.08) |
|  | Pleural effusion | 6 | 13.03(5.78-29.39) | 12.67(64.59) | 3.66(1.11) | 12.66(5.62) |
|  | Lung disorder | 5 | 11.11(4.57-27) | 10.85(44.8) | 3.44(0.79) | 10.85(4.46) |
|  | Obliterative bronchiolitis | 4 | 361.88(133.22-983) | 354.55(1383.93) | 8.44(0.92) | 347.94(128.09) |
|  | Tachypnoea | 4 | 36.61(13.59-98.62) | 35.88(135.46) | 5.16(0.78) | 35.82(13.29) |
|  | Aphonia | 3 | 17.48(5.59-54.7) | 17.23(45.86) | 4.11(0.16) | 17.21(5.5) |
|  | Atelectasis | 3 | 51.35(16.39-160.85) | 50.58(145.45) | 5.66(0.31) | 50.45(16.1) |
|  | Haemothorax | 3 | 203.23(64.59-639.49) | 200.15(588.18) | 7.63(0.37) | 198.03(62.93) |
|  | Hypercapnia | 3 | 114.54(36.5-359.48) | 112.81(330.5) | 6.81(0.36) | 112.14(35.73) |
|  | Obstructive airways disorder | 3 | 19.9(6.36-62.29) | 19.61(52.98) | 4.29(0.18) | 19.59(6.26) |
|  | Oropharyngeal oedema | 3 | 1157.23(357.66-3744.27) | 1139.62(3216.03) | 10.07(0.41) | 1073.93(331.92) |
|  | Pneumothorax | 3 | 24.94(7.97-78.07) | 24.58(67.81) | 4.62(0.22) | 24.55(7.84) |
|  | Pulmonary fibrosis | 3 | 19.44(6.21-60.83) | 19.16(51.61) | 4.26(0.18) | 19.14(6.12) |
| Renal and urinary disorders | Dysuria | 3 | 10.78(3.44-33.72) | 10.63(26.19) | 3.41(0.03) | 10.62(3.4) |
|  | Oliguria | 3 | 73.44(23.43-230.2) | 72.33(210.27) | 6.17(0.33) | 72.06(22.99) |
|  | Urinary retention | 3 | 14.11(4.51-44.16) | 13.91(35.96) | 3.8(0.1) | 13.9(4.44) |
| Psychiatric disorders | Hallucination | 25 | 49.31(32.4-75.04) | 43.18(1030.62) | 5.43(3.49) | 43.08(28.31) |
|  | Abnormal behaviour | 11 | 72.49(39.42-133.32) | 68.5(729.6) | 6.09(2.55) | 68.25(37.11) |
|  | Aggression | 9 | 45.27(23.17-88.42) | 43.24(370.93) | 5.43(2.14) | 43.15(22.09) |
|  | Confusional state | 9 | 7.06(3.62-13.79) | 6.79(44.68) | 2.76(1.17) | 6.78(3.47) |
|  | Agitation | 8 | 18.8(9.26-38.16) | 18.08(129.25) | 4.18(1.66) | 18.06(8.9) |
|  | Delirium | 5 | 21.74(8.94-52.84) | 21.21(96.29) | 4.41(1.04) | 21.19(8.71) |
|  | Hallucination, auditory | 5 | 43.6(17.92-106.05) | 42.52(202.36) | 5.41(1.18) | 42.42(17.44) |
|  | Fear | 4 | 21.93(8.14-59.04) | 21.5(78.17) | 4.42(0.68) | 21.48(7.97) |
|  | Nightmare | 4 | 16.45(6.11-44.29) | 16.13(56.8) | 4.01(0.61) | 16.12(5.99) |
|  | Sleep terror | 4 | 107.93(39.99-291.3) | 105.76(412.82) | 6.72(0.88) | 105.17(38.97) |
|  | Adjustment disorder | 3 | 242.31(76.92-763.36) | 238.64(700.99) | 7.88(0.38) | 235.63(74.8) |
|  | Anger | 3 | 14.42(4.61-45.14) | 14.22(36.88) | 3.83(0.11) | 14.21(4.54) |
|  | Catatonia | 3 | 84.87(27.07-266.13) | 83.6(243.77) | 6.38(0.34) | 83.23(26.54) |
|  | Delusion | 3 | 28.95(9.25-90.61) | 28.52(79.59) | 4.83(0.24) | 28.48(9.1) |
|  | Hallucination, visual | 3 | 18.32(5.85-57.33) | 18.05(48.32) | 4.17(0.17) | 18.04(5.76) |
|  | Mood swings | 3 | 12.48(3.99-39.04) | 12.3(31.17) | 3.62(0.07) | 12.3(3.93) |
|  | Poor quality sleep | 3 | 13.96(4.46-43.69) | 13.76(35.52) | 3.78(0.1) | 13.75(4.4) |
|  | Post-traumatic stress disorder | 3 | 36.4(11.63-113.99) | 35.86(101.53) | 5.16(0.27) | 35.8(11.43) |
|  | Psychotic disorder | 3 | 17.92(5.73-56.07) | 17.66(47.15) | 4.14(0.16) | 17.64(5.64) |
|  | Staring | 3 | 253.13(80.33-797.7) | 249.29(732.13) | 7.94(0.38) | 246.01(78.07) |
| Product issues | Product taste abnormal | 6 | 41.57(18.43-93.77) | 40.33(229.83) | 5.33(1.48) | 40.25(17.84) |
| Nervous system disorders | Seizure | 8 | 6.94(3.42-14.07) | 6.7(38.98) | 2.74(1.05) | 6.69(3.3) |
|  | Altered state of consciousness | 3 | 18.34(5.86-57.4) | 18.08(48.39) | 4.17(0.17) | 18.06(5.77) |
|  | Neuralgia | 3 | 10.16(3.25-31.8) | 10.03(24.4) | 3.32(0.01) | 10.02(3.2) |
| Musculoskeletal and connective tissue disorders | Muscular weakness | 5 | 5.03(2.07-12.23) | 4.93(15.74) | 2.3(0.32) | 4.93(2.03) |
|  | Compartment syndrome | 3 | 192.86(61.31-606.68) | 189.94(558.18) | 7.55(0.37) | 188.03(59.77) |
|  | Osteopenia | 3 | 17.95(5.73-56.16) | 17.69(47.23) | 4.14(0.16) | 17.67(5.65) |
| Metabolism and nutrition disorders | Dehydration | 12 | 11.59(6.46-20.78) | 10.94(108.97) | 3.45(1.83) | 10.94(6.1) |
|  | Hyperglycaemia | 3 | 10.94(3.5-34.24) | 10.79(26.67) | 3.43(0.03) | 10.78(3.45) |
|  | Hypernatraemia | 3 | 82.29(26.24-258) | 81.05(236.2) | 6.33(0.34) | 80.7(25.74) |
|  | Malnutrition | 3 | 35.05(11.2-109.75) | 34.53(97.55) | 5.11(0.27) | 34.47(11.01) |
| Investigations | Body temperature decreased | 4 | 33.43(12.41-90.05) | 32.77(123.07) | 5.03(0.76) | 32.72(12.14) |
|  | Influenza A virus test positive | 3 | 746.1(233.36-2385.45) | 734.76(2114.85) | 9.47(0.4) | 706.89(221.1) |
|  | Procalcitonin increased | 3 | 267.46(84.84-843.21) | 263.4(773.33) | 8.02(0.38) | 259.74(82.39) |
|  | Transaminases increased | 3 | 15.42(4.93-48.25) | 15.2(39.8) | 3.92(0.13) | 15.19(4.85) |
| Infections and infestations | Pneumonia | 11 | 3.94(2.15-7.24) | 3.78(22.81) | 1.92(0.77) | 3.78(2.06) |
|  | Influenza | 8 | 6.61(3.26-13.41) | 6.38(36.52) | 2.67(1.01) | 6.38(3.14) |
|  | Septic shock | 4 | 13.49(5.01-36.33) | 13.24(45.3) | 3.73(0.54) | 13.23(4.91) |
|  | Pneumonia necrotising | 3 | 1012.58(314.27-3262.56) | 997.17(2833.76) | 9.89(0.41) | 946.52(293.76) |
|  | Pneumonia streptococcal | 3 | 373.04(117.96-1179.74) | 367.38(1074.98) | 8.49(0.39) | 360.29(113.93) |
| Hepatobiliary disorders | Hepatitis cholestatic | 3 | 75.39(24.05-236.33) | 74.26(216) | 6.21(0.33) | 73.97(23.6) |
|  | Hepatosplenomegaly | 3 | 236.26(75.01-744.14) | 232.67(683.53) | 7.84(0.38) | 229.81(72.96) |
|  | Hyperbilirubinaemia | 3 | 50.43(16.1-157.98) | 49.68(142.77) | 5.63(0.31) | 49.55(15.82) |
| General disorders and administration site conditions | Crying | 6 | 18.83(8.35-42.46) | 18.29(98.12) | 4.19(1.27) | 18.27(8.1) |
|  | Multi-organ disorder | 5 | 381.95(155.83-936.15) | 372.28(1815.22) | 8.51(1.33) | 365(148.92) |
|  | Influenza like illness | 4 | 5.76(2.14-15.51) | 5.66(15.41) | 2.5(0.15) | 5.66(2.1) |
|  | Exercise tolerance decreased | 3 | 62.16(19.84-194.79) | 61.23(177.2) | 5.93(0.32) | 61.03(19.48) |
|  | Face oedema | 3 | 20.63(6.59-64.56) | 20.33(55.11) | 4.34(0.19) | 20.31(6.49) |
|  | Generalised oedema | 3 | 33.36(10.66-104.45) | 32.87(92.57) | 5.04(0.26) | 32.81(10.48) |
|  | Hypothermia | 3 | 38.9(12.42-121.82) | 38.33(108.88) | 5.26(0.28) | 38.25(12.21) |
| Gastrointestinal disorders | Vomiting | 41 | 10.79(7.65-15.22) | 8.75(288.14) | 3.13(2.42) | 8.75(6.2) |
|  | Haematemesis | 3 | 16.8(5.37-52.58) | 16.56(43.86) | 4.05(0.15) | 16.55(5.29) |
|  | Intestinal ischaemia | 3 | 86.16(27.48-270.19) | 84.87(247.55) | 6.4(0.34) | 84.49(26.94) |
|  | Mouth haemorrhage | 3 | 57.38(18.31-179.77) | 56.52(163.16) | 5.82(0.32) | 56.35(17.99) |
|  | Small intestinal obstruction | 3 | 31.28(9.99-97.92) | 30.82(86.45) | 4.94(0.25) | 30.77(9.83) |
|  | Toothache | 3 | 16.61(5.31-51.99) | 16.38(43.31) | 4.03(0.14) | 16.36(5.23) |
| Eye disorders | Blindness unilateral | 3 | 25.05(8-78.42) | 24.69(68.13) | 4.62(0.22) | 24.66(7.88) |
|  | Conjunctival haemorrhage | 3 | 107.18(34.16-336.3) | 105.56(309) | 6.71(0.35) | 104.97(33.45) |
|  | Symblepharon | 3 | 2577.49(765.17-8682.36) | 2538.26(6695.76) | 11.13(0.45) | 2233.78(663.13) |
| Ear and labyrinth disorders | Ear disorder | 3 | 58.81(18.77-184.26) | 57.93(167.36) | 5.85(0.32) | 57.75(18.43) |
|  | Ear inflammation | 3 | 708.8(221.93-2263.71) | 698.02(2012.64) | 9.39(0.4) | 672.83(210.67) |
| Cardiac disorders | Bradycardia | 5 | 12.21(5.02-29.69) | 11.93(50.14) | 3.58(0.83) | 11.92(4.9) |
|  | Myocarditis | 3 | 34.5(11.02-108.01) | 33.99(95.92) | 5.08(0.27) | 33.93(10.84) |
| Blood and lymphatic system disorders | Leukopenia | 10 | 22.99(12.17-43.45) | 21.88(199.44) | 4.45(2.05) | 21.85(11.56) |
|  | Lymphocytosis | 3 | 194.18(61.73-610.86) | 191.24(562) | 7.56(0.37) | 189.3(60.18) |

ROR, reporting odds ratio; CI, confidence interval; PRR, proportional reporting ratio; χ 2, chi-squared; IC, information component; EBGM, empirical Bayesian geometric mean; SOC, system organ class.

Supplementary Table 5. AE report of baloxavir marboxil as the primary suspect drug and oseltamivir as a co-administered drug.

| primaryid | age | age_cod | sex | rept_dt | role_cod | drugname | drug_seq | PT |
| --- | --- | --- | --- | --- | --- | --- | --- | --- |
| 159952624 | 15 | YR | F | 20190603 | PS | XOFLUZA | 1 | Anaphylactic reaction |
| 159952624 | 15 | YR | F | 20190603 | SS | CALONAL | 2 | Anaphylactic reaction |
| 159952624 | 15 | YR | F | 20190603 | C | RELENZA | 3 | Anaphylactic reaction |
| 159952624 | 15 | YR | F | 20190603 | C | TAMIFLU | 4 | Anaphylactic reaction |
| 222311391 | 6 | YR | F | 20230420 | PS | XOFLUZA | 1 | Confusional state |
| 222311391 | 6 | YR | F | 20230420 | SS | TAMIFLU | 2 | Confusional state |
| 222314941 | 11 | YR | F | 20230420 | PS | XOFLUZA | 1 | Off label use |
| 222314941 | 11 | YR | F | 20230420 | PS | XOFLUZA | 1 | Drug hypersensitivity |
| 222314941 | 11 | YR | F | 20230420 | SS | TAMIFLU | 2 | Off label use |
| 222314941 | 11 | YR | F | 20230420 | SS | TAMIFLU | 2 | Drug hypersensitivity |

AE, adverse event; YR, year; F, female; PS, primary suspect drug; SS, secondary suspect drug; C, concomitant; PT, preferred term.

Supplementary Table 6. AE report of oseltamivir as the primary suspect drug and baloxavir marboxil as a co-administered drug.

| primaryid | age | age_cod | sex | rept_dt | role_cod | drugname | drug_seq | pt |
| --- | --- | --- | --- | --- | --- | --- | --- | --- |
| 185354601 | 12 | YR |  | 20201123 | PS | TAMIFLU | 1 | Hallucination |
| 185354601 | 12 | YR |  | 20201123 | PS | TAMIFLU | 1 | Medication error |
| 185354601 | 12 | YR |  | 20201123 | PS | TAMIFLU | 1 | Oropharyngeal pain |
| 185354601 | 12 | YR |  | 20201123 | PS | TAMIFLU | 1 | Product administration error |
| 185354601 | 12 | YR |  | 20201123 | PS | TAMIFLU | 1 | Pyrexia |
| 185354601 | 12 | YR |  | 20201123 | SS | XOFLUZA | 2 | Hallucination |
| 185354601 | 12 | YR |  | 20201123 | SS | XOFLUZA | 2 | Medication error |
| 185354601 | 12 | YR |  | 20201123 | SS | XOFLUZA | 2 | Oropharyngeal pain |
| 185354601 | 12 | YR |  | 20201123 | SS | XOFLUZA | 2 | Product administration error |
| 185354601 | 12 | YR |  | 20201123 | SS | XOFLUZA | 2 | Pyrexia |
| 222047331 | 8 | YR | M | 20230413 | PS | TAMIFLU | 1 | Blood pressure decreased |
| 222047331 | 8 | YR | M | 20230413 | PS | TAMIFLU | 1 | Foaming at mouth |
| 222047331 | 8 | YR | M | 20230413 | PS | TAMIFLU | 1 | Vomiting |
| 222047331 | 8 | YR | M | 20230413 | PS | TAMIFLU | 1 | Syncope |
| 222047331 | 8 | YR | M | 20230413 | PS | TAMIFLU | 1 | Ill-defined disorder |
| 222047331 | 8 | YR | M | 20230413 | SS | OSELTAMIVIR PHOSPHATE | 2 | Blood pressure decreased |
| 222047331 | 8 | YR | M | 20230413 | SS | OSELTAMIVIR PHOSPHATE | 2 | Foaming at mouth |
| 222047331 | 8 | YR | M | 20230413 | SS | OSELTAMIVIR PHOSPHATE | 2 | Vomiting |
| 222047331 | 8 | YR | M | 20230413 | SS | OSELTAMIVIR PHOSPHATE | 2 | Syncope |
| 222047331 | 8 | YR | M | 20230413 | SS | OSELTAMIVIR PHOSPHATE | 2 | Ill-defined disorder |
| 222047331 | 8 | YR | M | 20230413 | C | BALOXAVIR MARBOXIL | 3 | Blood pressure decreased |
| 222047331 | 8 | YR | M | 20230413 | C | BALOXAVIR MARBOXIL | 3 | Foaming at mouth |
| 222047331 | 8 | YR | M | 20230413 | C | BALOXAVIR MARBOXIL | 3 | Vomiting |
| 222047331 | 8 | YR | M | 20230413 | C | BALOXAVIR MARBOXIL | 3 | Syncope |
| 222047331 | 8 | YR | M | 20230413 | C | BALOXAVIR MARBOXIL | 3 | Ill-defined disorder |
| 222047331 | 8 | YR | M | 20230413 | C | CEFACLOR | 4 | Blood pressure decreased |
| 222047331 | 8 | YR | M | 20230413 | C | CEFACLOR | 4 | Foaming at mouth |
| 222047331 | 8 | YR | M | 20230413 | C | CEFACLOR | 4 | Vomiting |
| 222047331 | 8 | YR | M | 20230413 | C | CEFACLOR | 4 | Syncope |
| 222047331 | 8 | YR | M | 20230413 | C | CEFACLOR | 4 | Ill-defined disorder |
| 222047331 | 8 | YR | M | 20230413 | C | SACCHAROMYCES CEREVISIAE | 5 | Blood pressure decreased |
| 222047331 | 8 | YR | M | 20230413 | C | SACCHAROMYCES CEREVISIAE | 5 | Foaming at mouth |
| 222047331 | 8 | YR | M | 20230413 | C | SACCHAROMYCES CEREVISIAE | 5 | Vomiting |
| 222047331 | 8 | YR | M | 20230413 | C | SACCHAROMYCES CEREVISIAE | 5 | Syncope |
| 222047331 | 8 | YR | M | 20230413 | C | SACCHAROMYCES CEREVISIAE | 5 | Ill-defined disorder |
| 222047331 | 8 | YR | M | 20230413 | C | IBUPROFEN | 6 | Blood pressure decreased |
| 222047331 | 8 | YR | M | 20230413 | C | IBUPROFEN | 6 | Foaming at mouth |
| 222047331 | 8 | YR | M | 20230413 | C | IBUPROFEN | 6 | Vomiting |
| 222047331 | 8 | YR | M | 20230413 | C | IBUPROFEN | 6 | Syncope |
| 222047331 | 8 | YR | M | 20230413 | C | IBUPROFEN | 6 | Ill-defined disorder |
| 222047331 | 8 | YR | M | 20230413 | C | AMBROXOL HYDROCHLORIDE;CLENBUTEROL HYDROCHLORIDE | 7 | Blood pressure decreased |
| 222047331 | 8 | YR | M | 20230413 | C | AMBROXOL HYDROCHLORIDE;CLENBUTEROL HYDROCHLORIDE | 7 | Foaming at mouth |
| 222047331 | 8 | YR | M | 20230413 | C | AMBROXOL HYDROCHLORIDE;CLENBUTEROL HYDROCHLORIDE | 7 | Vomiting |
| 222047331 | 8 | YR | M | 20230413 | C | AMBROXOL HYDROCHLORIDE;CLENBUTEROL HYDROCHLORIDE | 7 | Syncope |
| 222047331 | 8 | YR | M | 20230413 | C | AMBROXOL HYDROCHLORIDE;CLENBUTEROL HYDROCHLORIDE | 7 | Ill-defined disorder |

AE, adverse event; YR, year; M, male; PS, primary suspect drug; SS, secondary suspect drug; C, concomitant; PT, preferred term.
